# Supplementary material for: A Deep-Dream Virtual Reality Platform for Studying Altered Perceptual Phenomenology
Source: Sci Rep. 2017 Nov 22;7:15982. doi: 10.1038/s41598-017-16316-2 (PMC5700081; doi:10.1038/s41598-017-16316-2)
Supplement: Supplementary file 4 — Supplementary Material [file 41598_2017_16316_MOESM4_ESM.pdf]

Supplementary Material

A Deep-Dream Virtual Reality Platform for Studying Altered Perceptual Phenomenology

Keisuke Suzuki, Warrick Roseboom, David J. Schwartzman, Anil K. Seth

Video S1

The video was generated by selecting a higher DCNN layer that responds selectively to higher-level categorical features (layers = 'inception_4d/pool', octaves = 3, octave scale = 1.8, iterations = 32, jitter = 32, zoom = 1, step size = 1.5, blending ratio for optical flow = 0.9, blending ratio for background = 0.1). For more detail of the algorithm see our GitHub repository [1].

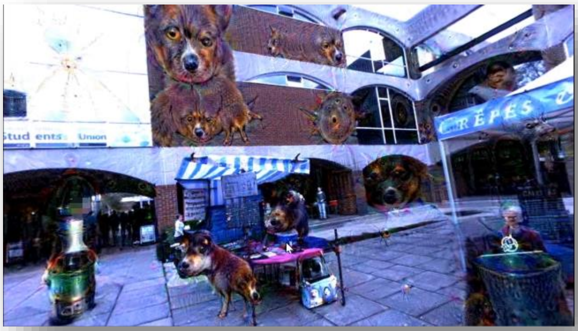

Video S2

The video was generated by fixing the activity of a lower DCNN layer that responds selectively to geometric image features (layer='conv2/3x3', octaves = 3, octave scale = 1.8, iterations = 32, jitter = 32, zoom = 1, step size = 1.5, blending ratio for optical flow = 0.9, blending ratio for background = 0.1). For more detail of the algorithm see our GitHub repository [1].

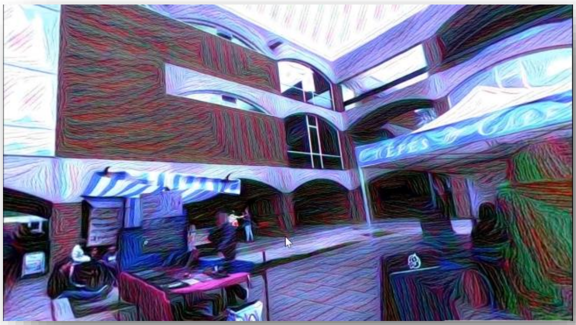

Video S3

The video was generated by selecting a middle DCNN layer responding selectively to parts of objects (layers = ''inception_3b/output'', octaves = 3, octave scale = 1.8, iterations = 32, jitter = 32, zoom = 1, step size = 1.5, blending ratio for optical flow = 0.9, blending ratio for background = 0.1). For more detail of the algorithm see our GitHub repository [1].

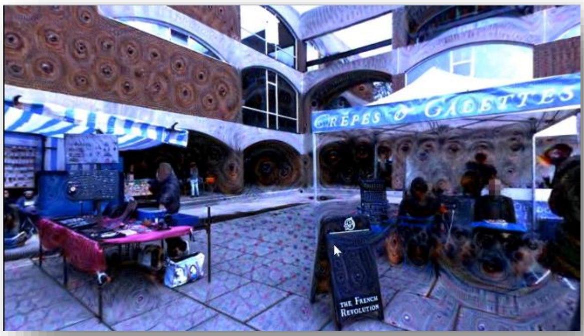

1. Ksk-S. DeepDreamVideoOpticalFlow (ksk-S/DeepDreamVideoOpticalFlow). *GitHub repository* (2017). Available at: https://github.com/ksk-S/DeepDreamVideoOpticalFlow.
